# 

**Published:** 1877-07

**Authors:** 


MCDOWELL MEDICAL SOCIETY.—Sixth annual session, 1877. Address by the President, E. H. Luckett, M.D., of Owensboro, Ky. Read at Madisonville, Ky., May 9, 1877.

CALCULI FOUND IN THE BLADDER AFTER THE CURE OF VESICO-VAGINAL FISTULA.— By Henry F. Campbell, M.D., Augusta, Ga. Reprint from Vol. 1, Gynæcological Transactions. 1876.

PNEUMATIC SELF-REPLACEMENT OF THE GRAVID AND NON-GRAVID UTERUS.—By Henry F. Campbell; Augusta, Ga. Reprint from Vol. I, Gynæcological Transactions, 1876.

HISTORY OF A CASE OF RECURRING SARCOMATOUS TUMOR OF THE ORBIT IN A CHILD.—Extirpated the third time, and ultimately causing death of the patient. By Thomas Hay, M.D., Philadelphia. Illustrated. 1877.

